# Zinc Phosphide Poisoning

**DOI:** 10.1155/2014/589712

**Published:** 2014-06-30

**Authors:** Erdal Doğan, Abdulmenap Güzel, Taner Çiftçi, İlker Aycan, Feyzi Çelik, Bedri Çetin, Gönül Ölmez Kavak

**Affiliations:** Department of Anesthesiology and Reanimation, Dicle University Medical School, 21280 Diyarbakir, Turkey

## Abstract

Zinc phosphide has been used widely as a rodenticide. Upon ingestion, it gets converted to phosphine gas in the body, which is subsequently absorbed into the bloodstream through the stomach and the intestines and gets captured by the liver and the lungs. Phosphine gas produces various metabolic and nonmetabolic toxic effects. Clinical symptoms are circulatory collapse, hypotension, shock symptoms, myocarditis, pericarditis, acute pulmonary edema, and congestive heart failure. In this case presentation, we aim to present the intensive care process and treatment resistance of a patient who ingested zinc phosphide for suicide purposes.

## 1. Introduction 

Zinc phosphide is a dark grey, crystalline compound. It is used as a rodenticide against such small mammals as mice, rats, field mice, and squirrels [1–4].

It is possible to be exposed to zinc phosphide poisoning by accident or through suicide. Once ingested into the body it transforms into phosphine gas and then with the help of the stomach and intestines mixes into the blood and is caught up by the liver and lungs. There are no antidotes currently known. The mortality rate of zinc phosphide poisoning is around 37–100% [5].

Organophosphate poisonings such as zinc phosphide poisoning are a significant cause of morbidity and mortality among socioeconomically low and economically active age demographics, especially in developing countries.

A 21-year-old subject is presented in this case who ingested zinc phosphide in order to commit suicide.

## 2. Case

A 21-old-year female ingested 6 grams of zinc phosphide mixed with water in order to commit suicide. The patient underwent gastric flushing and activated charcoal treatment at the county government hospital where she was admitted. The patient was dispatched from here to the university hospital; she was treated in the emergency ward. She was conscious, tending toward sleep, GKS 12, lung sounds natural, blood pressure 100/60 mmHg, and pulse 82 per minute. As the patient was evaluated in the emergency ward and subject to the initial intervention procedures, the consent form was signed by the patient's relatives, and the patient was taken to the intensive care unit. The patient's hemodynamic data was as follows: blood pressure 90/60 mmHg, SpO2 97, and pulse 85/m. Activated charcoal application at 2 mg/kg was continued, and supportive therapy was initiated. To improve the patient's urine output, furosemide was started after the fluid replacement IV. The patient was administered at a rate of 3 L/m oxygen through a mask. Full blood count, biochemistry, coagulation parameters, and arterial blood gas (ABG) readings of the patient were normal, with urine output at 0.5 mL/kg/hour, and the patient was hemodynamically stable. After receiving intensive care for 6 hours, the patient started to experience agitations and respiratory distress and developed resistant hypotension that was unresponsive to liquid replacement. With a normal AKG level, the patient was started on a 5 mcg/kg/minute dopamine infusion. Following increased respiratory distress, the patient was intubated and linked to the mechanical ventilator with a SIMV mode frequency of 12, tidal volume 500 mL, fio2 40%, peep 5 cm, and H_2_O linked. The patient was started on a midazolam infusion at a rate of 0.1 mg/kg/hour. The patient had no secretions during her in-tube aspiration, but about two hours after the intubation she began to aspire a bloody and bubbly secretion inside the tube. Thereupon the lung edema treatment was initiated on the patient. 10 hours after being taken into intensive care, the patient developed metabolic acidosis and, after being administered a 70 mEq bicarbonate replacement, was started on a 25 mEq/h infusion. According to the blood gas values, the metabolic acidosis table did not improve, even though it was interrupted at clear intervals with bicarbonate. Despite the patient having the dopamine infusion dose increased to 20 mcg/kg/minute as the hypotension deepened, the hypotension levels were not corrected, and the nonadrenaline infusion was started at a dose of 0.5 mcg/kg and then increased in a gradual fashion. Hemodialysis was considered for the patient, but due to the impaired hemodynamics (hypotension) the hemodialysis was not applied to the patient. Despite all the treatments the hemodynamics of the patient did not improve, and as the metabolic acidosis increased and the patient was being prepared in the ICU for bedside hemodiafiltration, the patient went into cardiac arrest and expired after cardiopulmonary resuscitation was applied for 45 minutes.

## 3. Discussion

Phosphide is used widely by young and productive members of society in suicide attempts [6]. In another study, the average age of patients who attempted suicide was reported to be 27 years [7]. The characteristics of our case fit the patient profile in the literature.

Zinc phosphide's mechanism of action upon oral ingestion is unclear. Possibly phosphine gas forms in the stomach after oral intake of zinc phosphide. Phosphine is rapidly absorbed, and upon the inhibition of C oxidase, mitochondrial morphology and oxidative respiration are impaired at a cellular level. Due to the extent of damage to the heart and the lungs, the patients are lost in the early stages [1, 5, 8, 9].

Phosphine gas causes various metabolic and nonmetabolic toxic effects. Clinical symptoms are circulatory collapse, hypotension, shock symptoms, myocarditis, pericarditis, acute pulmonary edema, and congestive heart failure [10]. In addition, gastrointestinal symptoms (nausea, vomiting, and diarrhea), hepatomegaly, severe metabolic acidosis, and acute kidney failure are observed in patients. Nausea, vomiting, diarrhea, retrosternal pain, shortness of breath, and cyanosis can be named among other symptoms. Also, hepatomegaly, liver failure, severe hypoglycaemia, delirium, tonic-clonic seizures, and acute severe metabolic acidosis (distal renal tubular acidosis) can be seen in these patients [5, 10–12]. Chugh et al. reported in their studies that shock, oliguria, coma, and convulsions could develop, and pulmonary edema, metabolic acidosis, hypocalcaemia, hepatotoxicity, and thrombocytopenia could be seen in cases of zinc phosphide poisoning [13]. In our patient, zinc phosphide poisoning-related circulatory collapse and lung damage developed. At the same time, the very severe hypotension and resistant metabolic acidosis that did not respond to bicarbonate treatment have been found to be in line with the literature. In cases of phosphide poisoning hypotension is a common occurrence, may develop quickly, and may be resistant to treatment.

In patients poisoned with Karanth and Nayyar rodenticide, severe hepatic dysfunction has been reported [14]. Frangides et al. have reported that after phosphide ingestion, temporary increases in alanine aminotransferase (ALT) and aspartate aminotransferase (AST) values are not infrequent [15–18]. In our case, although a slight increase in ALT occurred, no changes occurred in the AST values.

Pulmonary edema is commonly observed, but its etiology cannot be fully explained. Usually 4–48 hours following oral ingestion of zinc phosphide, PaCO_2_ decreases without an increase in the pulmonary artery pressure. ARDS-related pulmonary edema and nonspecific pulmonary edema are observed. Edema fluid may be protein-rich and haemorrhagic [11, 12, 19]. After undergoing intensive care for approximately 9 hours, our patient developed an acute pulmonary edema. According to the analysis of the bedside echocardiogram, the patient had no myocardial, pericardial, or cardiac pulse. After 10 hours of intensive care, the patient developed treatment-resistant hypotension despite being supported by a high dose of inotrope. Within a very short time prior to the patient passing away, a definitive pulmonary edema diagnosis was not possible.

## 4. Conclusion

Zinc phosphide is a substance that causes life-threatening complications. Unfortunately, there is neither an antidote, nor a specific treatment for it. Despite a quick and aggressive supportive therapy, heart or lung damage due to zinc phosphide poisoning is associated with a quite high mortality risk.
